# Diseases of Circulatory System

**Published:** 1905-03-18

**Authors:** 


### Progress in Medicine and Surgery.

### DISEASES OF CIRCULATORY SYSTEM.

**Arterio-Sclerosis.**—Osler[1] in speaking of angina pectoris notices that degeneration of the coronary vessels is not a complete explanation of the pain. There are four types of angina: (*a*) the neurotic, in which young persons suffer and even die, and yet the coronary arteries and the systemic vessels show no trace of disease; (*b*) the syphilitic with lesions in the coronary vessels, the root of the aorta, or the sigmoid valves. Young men are affected and may be cured by iodides, or develop aneurysms of the aorta; (*c*) the ordinary presenile type with coronary and other scleroses. Still angina occurs in only a few cases of coronary sclerosis; (*d*) the senile type which is not uncommon after 70 as the end of degeneration. Pain, indeed, is found with other lesions of vessels, such as the headache of high tension, and the severe pain in some cases of cerebral embolism, and lesions of the mesenteric vessels. Again in sclerosis of the arteries of the limbs we may get numbness and tingling, painful cramps, and paroxysmal pain with erythromelalgia. Thayer[2] shows that the infective fevers often give rise to arterio-sclerosis, and that after an attack of typhoid a large number of patients suffer from it. Among other important causes are syphilis, scarlatina, rheumatism, alcohol, and hard labour. Cabot investigated the influence of alcohol in a large number of cases and everywhere found that it is of little importance as a cause. Thayer acknowledges that it does far less than prolonged physical over-exertion.

**Blood Pressure.**—J. M. Cowan[3] in an account of recent work, notices that pressure increases during growth and may be high in obesity. The average in healthy adults is from 105 to 115 mm. In women it is a little lower than in men of the same age, but is increased at puberty, menstruation, and the menopause, though not in pregnancy. Potain thinks the pressure varies in different vessels, but Oliver and von Recklinghausen deny this. No alteration in pressure is produced by change in the volume of the blood unless this is great. Bleeding to 16 oz. will often leave it unaltered, and saline infusions only raise it when they are large. Position is important, the raising of the head by a pillow causes a variation of 1 cm. in the temporal artery, and the lifting of a leg alters the pressure in the whole body. Moderate exercise raises, and fatigue lowers it; regular exercise tends to a lower general pressure. There is a temporary rise after swallowing food or drink, but then follows a gradual fall for some hours, cerebral excitement raises, and a sudden rise of atmospheric pressure causes a temporary fall. Phthisis leads to a steady fall, except during fever or complications, and this alone distinguishes the disease from chlorosis except when in the latter the heart is dilated. In enteric fever the steady fall of pressure is only interrupted if perforation or peritonitis occurs, and this rise is a valuable symptom. In pneumonia and acute rheumatism pressures, as a rule, are lowered, and this, too, is the case in many cases of failing compensation in cardiac disease, but in others there is a rise from the effects of œdema and toxæmia. There is, of course, often a rise in Bright's disease and in the paroxysms of angina. In atheroma, if the large vessels are chiefly affected, the rise is not so great as when the peripheral ones generally are attacked. The latter condition may be due to previous attacks of scarlatina, typhoid, and gouty complaints. Allbutt has postulated an increased viscosity of the blood as the frequent cause of high pressure in later middle age, which is probably due to over-feeding. Certain cases of high pressure in renal disease have been found to have hyperplasia of the suprarenals, and this was absent in others where the pressure was low, but the observations are open to doubt. Potain claims high pressure for diabetes. It occurs, too, in mania, and generally in epileptics. Crile thinks that the essential feature in surgical shock is a fall of blood pressure due to exhaustion of the vaso-motor centres. On the other hand collapse is due to suspension of function of the heart or vaso-motor mechanism, and here stimulants, saline injections, and strychnine are useful, but in shock they are of little value. The only thing which will raise pressure in shock is general peripheral pressure for which he has employed an indiarubber suit to compress the surface of the body. The effect of adrenalin in these cases is too transitory to be of use. A fall of pressure often occurs in narcosis, in operations on the genital organs, on the nervous system, or on the upper part of the abdomen, or after the removal of large tumours or large collections of fluid. Crile[4] allows that in normal animals a dose of strychnine sufficient to increase the reflexes produces a rise of pressure, and this is still higher if enough to cause convulsions is given, but continued high doses end in a fall of the pressure. R. C. Cabot[5] has made 5,C00 observations on febrile patients, and found that strychnine in amounts equal to one-eighth grain daily, either by the mouth or subcutaneously, causes no rise in the pressure. The same is true of alcohol, but mental excitement, or the prospect of food, causes a transient rise. Abrams,[6] in discussing the measurement of blood pressure, points out that we can eliminate one of the two factors which produce it, viz., vaso-constriction, by giving nitrite of amyl, and if we intend to administer cardiac tonics rationally this manœuvre, followed by the use of the manometer and auscultation, is the ideal procedure. Otherwise we cannot judge of the real source of the pressure and the strength of the heart.

Coagulation.—Boggs[7] investigated the effect of various injections on the coagulation time of the blood. Gelatin or the serum of other animals in guinea pigs gave uncertain results, extracts of liver and thymus decreased it, calcium chloride caused a reduction lasting two or three hours, and this reduction was obtained in human beings when it was given by the mouth. Donath in a study of paroxysmal hæmoglobinuria, found that the red cells do not show any marked susceptibility to cold, nor in any great degree to mechanical injury, neither has the serum when chilled a greater power of dissolving them. The red cells are not unusually subject to hæmolytic influences, such as serum from other individuals. He has failed to find a hæmolysin in the blood of these patients, but he believes that one is present and is the cause of the blood disintegration. Lancereaux[8] reports eight more cases of aneurysm of the aorta treated by gelatin injections. In all the pain was lessened or removed, and six patients improved. He employed a $2\frac{1}{2}$ per cent. solution of gelatin in normal saline solution, and injected 7 oz. every third or fourth day. The solution should be sterilised in steam, under pressure at 115° for 30 minutes, to destroy the organisms of tetanus which are common in gelatine. Cobbledick[9] describes a case of thrombosis of the superior vena cava in a woman aged 42. There was œdema of the face, the right side of the neck, and the right arm and hand, and also of the legs up to the knees. The left innominate vein showed a recent softening thrombus, and the right a firm one passing down into the superior vena cava as far as the opening of the vena azygos major, almost filling up the lumen. The anterior wall of the vessel was thickened, and there was some thickening of the tissues between it and the trachea. The condition was not due to external causes in this case, but such instances of simple phlebitis in the superior vena cava are extremely rare. Osler only met with three in the literature of the subject.

[1] Lancet, Oct. 29. [2] Med. Rec., p. 985, vol. i. [3] Practit., p. 222, vol. ii. [4] New York Med. Jour., Sept. 24. [5] Boston Med. and Surg. Jour., Sept. 29. [6] Amer. Jour. Med. Sci., Nov. [7] Ibid., Aug. [8] Edin. Med. Jour., Oct. [9] Practit, Nov.

*(To be continued.)*

